# Biliary hamartoma: a report of two cases

**DOI:** 10.11604/pamj.2022.42.57.29748

**Published:** 2022-05-19

**Authors:** Anass Haloui, Nassira Kariche, Asmae Aissaoui, Youness Najjioui, El Mehdi Tiabi, Noura Seghrouchni, Amal Bennani

**Affiliations:** 1 Laboratory of Pathological Anatomy, Faculty of Medicine and Pharmacy of Oujda, Mohammed First University, Oujda, Morocco

**Keywords:** Biliary hamartoma, Von Meyenburg complexes, ductal plate malformation, adenocarcinoma, case report

## Abstract

Biliary hamartoma, also known as biliary micro hamartoma or Von Meyenburg complex, is a rare benign liver lesion, thought to be a ductal plate malformation rather than a true neoplasm. It is often seen incidentally on imagery or surgery as multiple small subcapsular nodules, scattered throughout the liver, making it likely to be mistaken for metastatic nodules. The histological presentation can also be deceptive, leading to the misdiagnosis of an adenocarcinoma of hepato-biliary differentiation or a metastasis. We hereby present two cases of biliary hamartoma, found incidentally on imagery and surgery, the first one in a 94-year-old woman, and the second in a 48-year-old man, which was initially misdiagnosed as an adenocarcinoma, along with a discussion of key clinical and pathological findings to help avoid this diagnostic pitfall.

## Introduction

Biliary hamartoma is a rare benign liver lesion, described for the first time in 918 by Von Meyenburg [1]. It is thought to be secondary to congenital malformations of ductal plates rather than a true neoplasm, due to interrupted remodeling of ductal plates during the late phase of embryological development of small intrahepatic bile ducts [2]. The main diagnostic pitfall is to confuse these benign lesions with cholangiocarcinoma or metastatic adenocarcinoma. We herein report two cases of biliary hamartoma, one of which had initially been misdiagnosed as an adenocarcinoma prior to our proofreading, along with a discussion of the pathological features of relevance to a reliable diagnosis.

## Patient and observation

### Patient 1

**Patient information:** a 94-year-old woman, non-smoker, non-alcoholic, with no medical or surgical history, who presented abdominal pain.

**Clinical findings:** clinical examination revealed tenderness in the right hypochondriac region, with no palpable mass. Ultrasonography of the abdomen revealed a cephalic mass of the pancreas. In July 2020, the patient underwent a placement of a biliodigestive bypass with cholecystectomy, along with a biopsy of a suspicious hepatic nodule, which was found incidentally during the surgical procedure.

**Figure 1:**
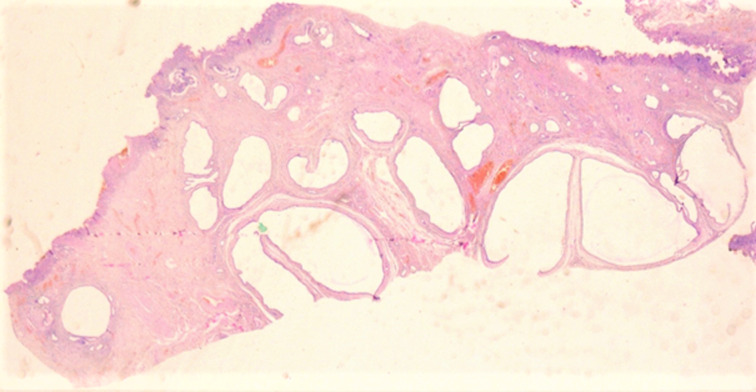
multiple dilated ducts embedded in a fibrous stroma

**Figure 2:**
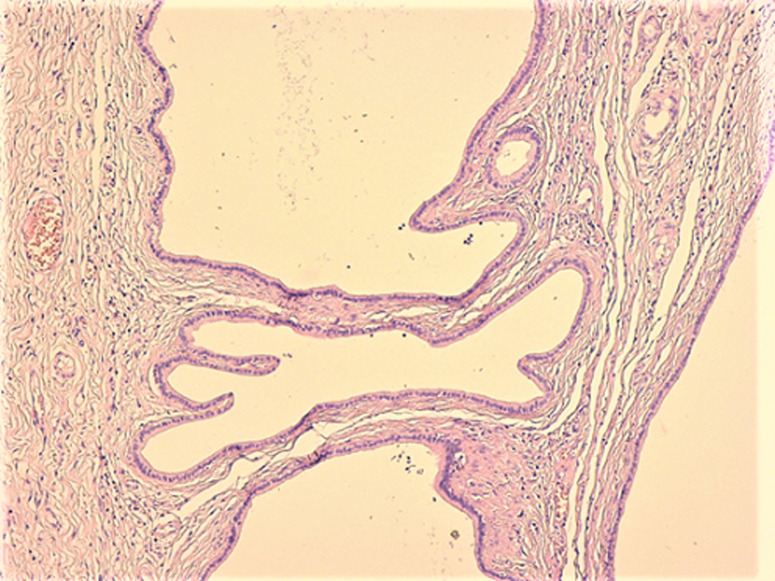
irregularly shaped ducts lined by a single layer of bland cuboidal epithelium

**Diagnostic assessment:** the histological examination of the hepatic nodule revealed a collection of numerous dilated and cystic ductal structures embedded in a dense fibrous stroma, lined by a single layer of cuboidal epithelium (Figure 1, Figure 2). The cells were not atypical, showing round nuclei with homogeneous chromatin and eosinophilic cytoplasm. Mitotic figures are absent. Focal inspissated bile was noticed within the lumina of the ductal structures.

**Diagnosis:** the results were consistent with a biliary hamartoma, based on morphological appearance.

**Therapeutic interventions:** the liver nodule was not surgically removed.

**Follow-up and outcome of interventions:** to date, the patient remains asymptomatic and in excellent clinical condition, with no reported complications.

**Patient perspective:** “I feel good”.

**Informed consent:** the patient gave informed consent.

### Patient 2

**Patient information:** a 48-year-old man, non-smoker, non-alcoholic, with no medical or surgical history, who presented abdominal pain.

**Clinical findings:** clinical examination revealed tenderness in the right hypochondriac region, with no palpable mass.

**Timeline of current episode:** an ultrasonography of the abdomen was performed revealing a liver nodule. A biopsy along with initial histological and immunohistochemical study were conducted, concluding to a metastatic adenocarcinoma, showing a diffuse expression of cytokeratin 7 (CK7), without expression of cytokeratin 20 (CK20), transcription termination factor 1 (TTF1) and prostate-specific antigen (PSA). The Paraffin embedded tissue blocks were addressed to our laboratory for proofreading.

**Figure 3:**
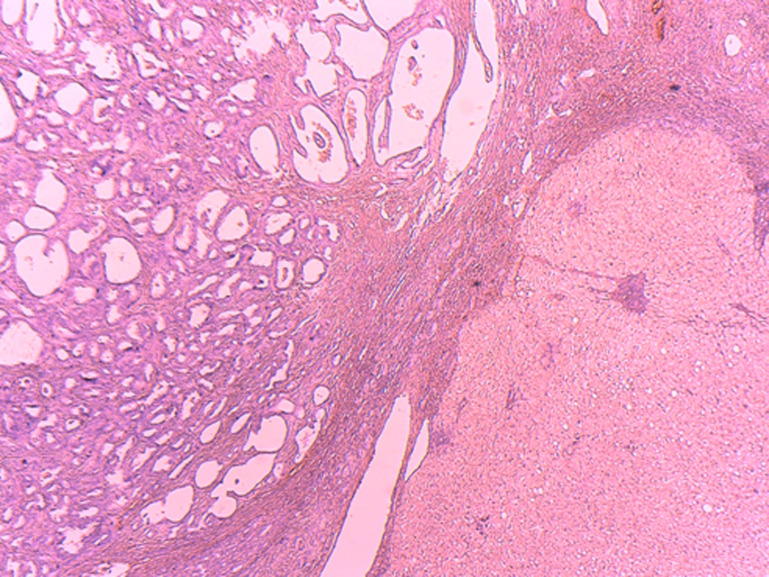
sharply limited lesion composed of multiple ducts adjacent to a normal hepatic tissue

**Figure 4:**
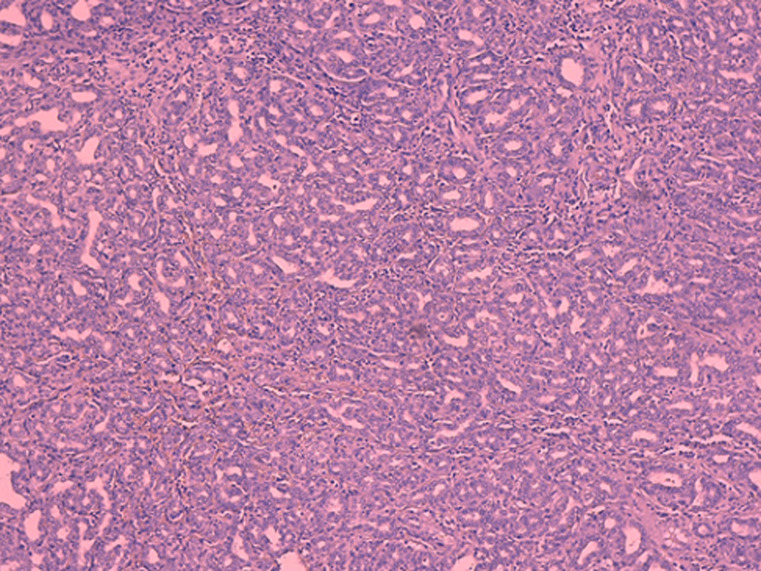
ducts of variable shapes and sizes, often rounded, embedded in a fibrous stroma

**Figure 5:**
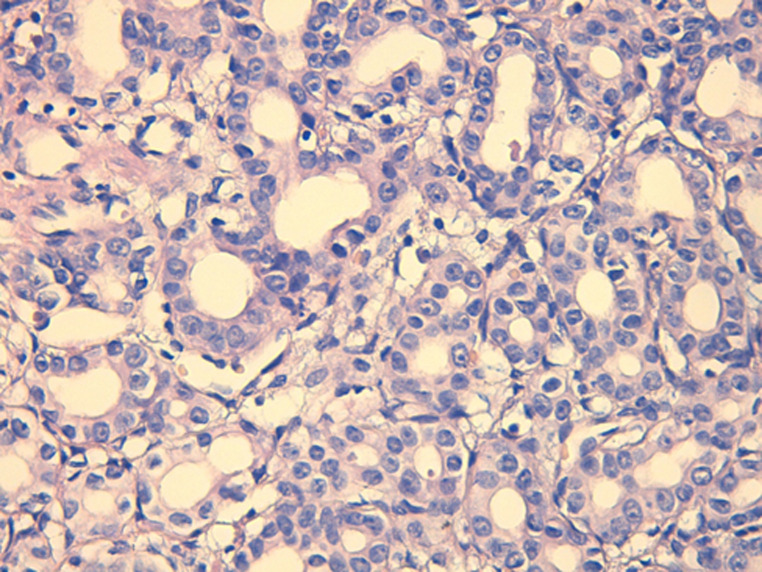
single layer of cuboidal epithelium, cellular atypia and mitotic figures are absent, focal inspissated bile can be seen

**Diagnostic assessment:** the histological examination of the tissue blocks revealed a well delimited lesion (Figure 3), composed of multiple duct-like structures, disposed in a fibrous stroma. The duct structures are of small to medium size, irregularly shaped or rounded (Figure 4), lined by a single layer of bland-looking cuboidal epithelium. Mitotic figures were absent (Figure 5).

**Diagnosis:** the diagnosis of biliary hamartoma was based on the morphological appearance and the lack of malignancy´s evidence.

**Therapeutic interventions:** the liver nodule was not surgically removed.

**Follow-up and outcome of interventions:** to date, the patient remains asymptomatic and in excellent clinical condition, with no reported complications.

**Patient perspective:** “I feel well”.

**Informed consent:** the patient gave informed consent.

## Discussion

Several different terms have been used to refer to biliary hamartoma, the most common are biliary micro hamartoma and Von Meyenburg complexes [3]. They can be seen sporadically, or commonly associated with fibrocystic liver or kidney disease, congenital hepatic fibrosis and Caroli disease [4]. A variant characterized by enlargement and cystic dilation of the biliary channels has been called Multicystic biliary hamartoma [5]. They occur three times more frequently in women than in men [6], with an estimated incidence of 5.6% in adults and 0.9% in children [7]. Most patients are asymptomatic. However, rare cases can cause abdominal pain, hemorrhage, cholangitis, jaundice, and portal hypertension. Although most of biliary hamartomas have some typical imaging features, radiology alone is usually non-diagnostic. On ultrasonography, the suggestive findings are a conglomerate of hypoechoic irregular masses with hyperechoic cystic walls. On CT scan, these lesions usually appear as low-density lesions with enhancing septas. On MRI, Von Meyenburg complexes shows low signal intensity on T1-weighted images, and high signal intensity on T2 weighted image [1].

On gross examination, biliary hamartoma appears as gray-white, cystic or solid, unique, or multiple nodules scattered throughout the liver. Generally, they measure less than 0.5 cm, although larger lesions up to 20 cm may be seen [8]. They are usually well-delineated and non-encapsulated, often portal or sub-capsular, making them visible to surgeons who may incidentally see them on the surface of the liver during abdominal surgery and submit a specimen for pathological examination to exclude metastatic disease [9], which was the case of our first patient. Histologically, biliary hamartomas consist of a circumscribed collection of inter-anastomosing ductal structures, embedded in a dense fibrous stroma. They are often located within or at the edge of portal tracts. The ducts are small to medium in size, irregular or rounded in shape, but may occasionally be dilated. They are lined with a single layer of cuboidal, columnar, or flattened epithelium and their lumens may contain eosinophilic debris or inspired bile. Cellular atypia and mitotic figures should be absent [10]. The stroma between the bile duct-like structures can be densely collagenous or less frequently loose and myxoid. Mild lymphocytic inflammation is also common. Immunohistochemically, the cells surrounding the ducts express cytokeratin 19 (CK19) and CK7, with a low proliferative rate on Ki 67. These features are consistent with histological findings in both of our cases.

While biliary hamartoma and biliary adenoma may have deceptive similarities in histological appearance, especially in the setting of small, rounded ducts, a careful evaluation of the morphological features is usually helpful in distinguishing between them. Morphological features favoring biliary hamartoma over biliary adenoma are more abundant and mature connective tissue, with greater variation in lumen size and shape. Furthermore, inspissated bile is common in biliary hamartomas, but not in bile duct adenomas (Table 1). In our first case, the liver nodule was made of dilated ducts of variable size and irregular shapes. Inspissated bile could also be seen focally. Therefore, the diagnosis of biliary hamartoma was made based on the morphological appearance.

**Table 1:** differential diagnosis of biliary hamartoma

Histological features	Biliary hamartoma	Biliary adenoma	Cholangiocarcinoma	Metastatic adenocarcinomas
**Biliary structures architecture**	Small, dilated tubules; inter anastomosing pattern of growth; open lumens; inspissated bile is common.	Generally small and round tubules; small or inapparent lumens; no inspissated bile.	Generally larger tumors; infiltrating borders and a destructive growth pattern.	Adenocarcinoma with variable appearance (although it is the well differentiated type that may resemble a biliary hamartoma).
**Cytology**	Cytologically bland biliary epithelium: no cytological atypia; no mitotic figures.	Bland cuboidal cells; regular nuclei, resembling ductules.	Cytological atypia mitotic figures, atypical mitosis cellular debris in the lumens of biliary structures (dirty luminal necrosis) strongly favors cholangiocarcinoma.	Cytological atypia; mitotic figures, atypical mitosis; 'garland-like' necrosis in cases of metastatic (colorectal adenocarcinomas).
**Stroma**	Loose and myxoid or densely collagenous; mild lymphocytic inflammation; most hamartomas are located adjacent to, or clearly involve, a portal tract.	Fibrous, shows varying degrees of chronic inflammation and collagenization; portal tracts are often enclosed in the nodule.	Fibrous stroma with marked inflammatory infiltrate; vascular or perineural invasion can be found.	Fibrous stroma with marked inflammatory infiltrate; vascular or perineural invasion can be found.
**Immunohistochemistry**	Express CK7 and CK19; low proliferative rate (Ki-67).	Express CK7 and CK19, and often MUC6, MUC5AC.	CK7, CK19, ACE: usually positive; CK20: positive in 20%; increased proliferation rate on Ki-67; P53: often strong expression.	Segregate according to CK7/CK20 pattern, then apply markers according to the suspected primary site.

The differential diagnosis also includes metastatic adenocarcinoma and cholangiocarcinoma, which tend to be larger in size, with an infiltrative growth pattern, in contrast to the small size and well limited character of hamartomas (Table 1). The liver nodule in our second patient was initially misdiagnosed as a liver metastasis of an adenocarcinoma expressing CK7. One can readily understand the reason for this confusion since a well-differentiated adenocarcinoma may very well display a tubular architecture, with tubes lined with cells that can show discrete atypia, which may be confused with the tubular structures usually present in biliary hamartomas. But in case of malignancy, it is classical to find a strong mitotic activity, atypical mitosis, necrosis, as well as a stromal reaction with an important inflammatory infiltrate. On our proofreading, all features suggestive of malignancy were absent. Moreover, the well limited character of the lesion, the presence of ducts with rounded or irregular lumen and the absence of cytological atypia allowed us to make the diagnosis of biliary hamartoma. Biliary hamartomas are not considered premalignant, although rare cases of cholangiocarcinoma arising in biliary hamartoma have been reported. The evolution is benign with no treatment required when asymptomatic.

## Conclusion

Biliary hamartomas are rare benign liver lesions, which may have misleading radiological and morphological features leading to a misdiagnosis of cholangiocarcinoma or metastatic adenocarcinoma. Therefore, careful evaluation of the morphologic appearance is essential to avoid misdiagnosis of a malignant lesion.
